# Completion *vs*. early discontinuation of chemotherapy and the impact on 5‐year all‐cause mortality in women treated for early‐stage breast cancer from 2015 to 2020: A cohort study using a target trial emulation approach

**DOI:** 10.1002/bcp.70488

**Published:** 2026-03-08

**Authors:** Luke Steventon, Chengsheng Ju, Kenneth K. C. Man, Rebecca Roylance, Diego Ottaviani, Chiara Creed, Michal Sladkowski, Alec Wisner, Li Wei, Pinkie Chambers

**Affiliations:** ^1^ UCL School of Pharmacy London UK; ^2^ Centre for Medicines Optimisation Research and Education (CMORE) London UK; ^3^ University College London Hospital NHS Foundation Trust London UK; ^4^ Department of Oncology UCL Cancer Institute London UK; ^5^ York and Scarborough Teaching Hospitals NHS Foundation Trust York UK; ^6^ Cleveland Clinic Cleveland Ohio USA; ^7^ UNC Eshelman School of Pharmacy University of North Carolina at Chapel Hill Chapel Hill North Carolina USA

**Keywords:** breast cancer, chemotherapy, survival outcomes, treatment discontinuation

## Abstract

**Aim:**

Chemotherapy is given for early‐stage breast cancer; however, some patients discontinue before completing all planned cycles. This study investigated the impact of early chemotherapy discontinuation on treatment outcomes.

**Methods:**

This retrospective cohort study used a target trial emulation framework to conduct a causal analysis of the all‐cause mortality impact of completing a standard course of chemotherapy. Early‐stage breast cancer patients treated with chemotherapy in England between 01/01/2014 and 31/12/2015 were identified from the National Disease Registration Service and Systemic Anti‐Cancer Therapy datasets. Five‐year OS was estimated for patients completing greater than or equal to six chemotherapy cycles relative to discontinuing chemotherapy early (less than six cycles), representing standard treatment during the study period. A clone‐censor‐weight approach was used to account for time‐related bias and baseline confounding. Absolute risk and hazard ratios (HRs) were calculated.

**Results:**

A total of 10 253 patients were included: 68% (*n* = 7014) received greater than or equal to six chemotherapy cycles, and 32% (*n* = 3239) received fewer than six cycles. Individuals completing greater than or equal to six cycles showed superior 5‐year OS compared with discontinuation less than six cycles (absolute risk difference −1.6, 95% confidence interval [CI], −3.2, −0.1; HR 0.85, 95% CI 0.74, 0.98). Subgroup analyses showed OS benefit in patients diagnosed at stage 2 relative to Stage 3 (HR 0.82, 95% CI 0.69, 0.98), ER+/HER2+ histology (HR 0.46, 95% CI 0.24, 0.96) and Non‐White ethnicity (HR 0.56, 95% CI 0.34, 0.91) when receiving six cycles.

**Conclusion:**

Patients who completed greater than or equal to six cycles showed improved OS compared with those who discontinued before receiving six cycles. These findings support the identification and pre‐emptive management of patients at high risk of discontinuing chemotherapy prematurely, to maximize treatment benefit.

**What is already known about this subject**

- Early chemotherapy discontinuation has been associated with reduced survival in several cancer types.
- However, the impact of early chemotherapy discontinuation in early‐stage breast cancer is poorly understood.

**What this study adds**

- This study is the first to use a target trial emulation study design to analyse the impact of early chemotherapy discontinuation.
- Early chemotherapy discontinuation was more frequent in patients of older age and those with higher body mass index.
- Women who discontinued chemotherapy before receiving six cycles showed significantly reduced 5‐year survival.

## 1 INTRODUCTION

Breast cancer is the most common cancer type, with a global incidence of 2.3 million in 2020.^1^ Although research and drug development have advanced therapies to optimize treatment outcomes, 16% of all cancer deaths in women are still attributed to breast cancer.^1^ In the United Kingdom, incidence is around 55 000 cases per year, comprising 15% of cancer diagnoses.^2^, ^3^ Each year, 11 500 breast cancer deaths occur in the United Kingdom, accounting for 7% of all cancer‐related deaths.^2^

Current guideline‐directed therapy for breast cancer includes surgery, radiotherapy, cytotoxic chemotherapy, endocrine, targeted and immunotherapy.^4^, ^5^ Treatment depends on tumour stage, grade, hormone receptor status (oestrogen [ER], progesterone [PR] and human epidermal growth factor receptor 2 [HER2]), patient age, performance status and other factors. Although advances in treatment have led to more targeted treatment approaches, cytotoxic chemotherapy continues to play a significant role in breast cancer treatment, both in neo‐adjuvant and adjuvant settings, with systemic therapy most often utilized in high‐risk patients with node‐positive disease to reduce mortality.^6^ Standard chemotherapy treatment regimens include an anthracycline and a taxane, both of which have significant toxicity considerations.^4^, ^5^ Anthracyclines are associated with cumulative dose‐dependent cardiotoxicity^7^ and risk of second malignancy,^8^ whereas the dose‐limiting toxicities of taxanes are frequently neutropenia^7^ and neurotoxicity.^9^ Drug toxicity is a major contributor to dose reductions, delays and early treatment discontinuation.

Survival outcomes associated with early discontinuation of systemic therapy have been studied in other cancer types such as pancreatic^10^ and colorectal cancer^11^; however, there has been limited research exploring the association of early discontinuation of cytotoxic chemotherapy with survival outcomes in breast cancer. A prospective cohort study conducted in the United States found 11.9% of patients prematurely discontinued chemotherapy.^12^ Those with longer planned treatment durations (greater than four cycles) and those receiving regimens with greater toxicity profiles were more likely to discontinue treatment. In colorectal cancer, early discontinuation of systemic chemotherapy has been associated with reduced disease‐free survival and all‐cause mortality.^13^, ^14^, ^15^ These studies suggest the value of optimizing patient care to maximize the chance of successfully completing the full treatment course. There have been studies investigating the impact of early discontinuation of trastuzumab^16^, ^17^ and endocrine therapies^18^, ^19^ in retrospective analyses; however, the impact of early chemotherapy discontinuation on survival in early breast cancer has not been established.

This study is the first of its kind, using a sophisticated methodological approach to address a gap in knowledge of the impact of early chemotherapy discontinuation. This target trial emulation study examines the association between early discontinuation of cytotoxic chemotherapy and cancer‐related mortality in a historical cohort of patients treated with systemic therapy for early‐stage breast cancer (i.e., breast cancer that has not spread beyond the breast and/or the axillary lymph nodes).

## 2 METHODS

### 2.1 Data sources

The National Disease Registry Service (NCRD, or Cancer Registry) is a registry of all patients diagnosed with cancer in England, containing tumour‐specific information and baseline patient characteristics such as age, ethnicity, socio‐economic status and other demographic factors.^20^ The Systemic Anti‐Cancer Therapy (SACT) dataset provides records of SACT treatment, including drug names, doses and treatment dates. Death dates were provided by the Office for National Statistics for each patient in the cancer registry. The NCRD has recorded cancer diagnostic information since 1971 for patients in England, and reporting of systemic therapy information to the SACT dataset has been mandated since 2014.^21^ Data resource profiles have been published for both datasets, providing a detailed discussion of data quality and population coverage for NCRD^20^ and SACT datasets.^22^ These datasets provide coverage for all patients treated with SACT for cancer within the NHS, representing the vast majority of cancer patients in England, and can be linked using common pseudonymized patient identifiers, providing a powerful resource for epidemiological research.

### 2.2 Study design

This observational, retrospective cohort study followed the target trial emulation framework. A hypothetical target trial was designed to answer the causal question of interest: to compare 5‐year all‐cause mortality between ‘completion’ of greater than or equal to six cycles of chemotherapy or ‘early discontinuation’ (receiving less than six chemotherapy cycles). Standard of care treatment during the 2014–2020 study period was to receive six cycles of 3‐weekly chemotherapy; however, some clinicians may have chosen to give up to eight cycles, depending on the treatment regimen; therefore, we included patients who may have received up to eight cycles. Discussion of clinical practice during the study period with oncologist coauthors informed the treatment strategies compared in this analysis.

The target trial was then emulated using Cancer Registry and SACT datasets. The clone‐censor‐weight design was applied to investigate this research question and to account for time‐related biases common in conducting research using observational data, according to methodology previously used to investigate outcomes of treatment with chemotherapy.^23^ Specifications of the target trials and trial emulation are presented in Table 1. Further specification of the study design is given in the Supporting Information.

**TABLE 1:** Protocol summaries of the target trials and trial emulation.

Component	Target trial specification	Target trial emulation
Eligibility criteria	Adult patients ≥18 years diagnosed with early‐stage (Stage II/III) breast cancer in England and treated with chemotherapy as first‐line treatment between 1st January 2014 and 31st December 2015. Regimens for early‐stage breast cancer include epirubicin (E), cyclophosphamide (C), fluorouracil (F) and docetaxel (T). Drug regimens are EC, EC‐T/T‐EC and FEC‐T/T‐FEC.	Same as the target trial
Treatment strategies	Number of cycles of chemotherapy: greater than or equal to six cycles *vs*. less than six cycles Early discontinuation is defined as the last record of first‐line chemotherapy. A break of >63 days (3‐week interval plus a 6‐week grace period) between chemotherapy treatments was considered a new line of therapy.	Same as the target trial
Treatment assignment	Randomization	Randomization is emulated via the clone‐censor‐weight approach.
Outcomes	5‐year all‐cause mortality (from chemotherapy initiation)	Same as the target trial
Follow‐up	For each patient, the follow‐up starts at the first chemotherapy treatment and ends at death or last known follow‐up or end of 5‐year follow‐up.	Same as the target trial
Causal contrasts	Intention‐to‐treat and per protocol effects	Only observational analogue of per‐protocol effect was emulated.
Statistical analysis	Patients are censored when they deviate from the assigned treatment strategy, i.e., if they discontinue chemotherapy before receiving 6 cycles. Selection bias introduced by artificial censoring is accounted for by using inverse probability of censoring weights that are calculated based on measured covariates.	Same as the target trial Inverse probability of censoring weights is used to account for artificial censoring in the cloned patient cohorts. Same subgroup analyses

### 2.3 Study population

Female patients ≥18 years diagnosed with early‐stage (II/III) breast cancer and treated with curative intent with standard first‐line cytotoxic chemotherapy between 01/01/2014 and 31/12/2015 were eligible. Male patients represent <1% of breast cancer diagnoses, are often diagnosed at later stages and show different tumour hormone profiles^24^; therefore, to maintain a homogenous population for analysis, these patients were not included in the data request. Standard chemotherapy drugs used at the time of data collection include epirubicin (E), cyclophosphamide (C), fluorouracil (F) and docetaxel (T). Regimens include these drugs given in combination and/or sequentially: EC (epirubicin and cyclophosphamide), EC‐T/T‐EC (epirubicin, cyclophosphamide and docetaxel), FEC‐T/T‐FEC (fluorouracil, epirubicin, cyclophosphamide and docetaxel) for a total of six cycles.^25^, ^26^, ^27^ Docetaxel was used as the taxane of choice during the study period.

Patients treated with docetaxel and cyclophosphamide (TC) were excluded, as many clinicians would prescribe TC with four cycles of treatment planned; therefore, we could not reliably ascertain whether patients who received four cycles of TC had discontinued chemotherapy early or completed all planned cycles. Although we acknowledge that systemic therapies for breast cancer have evolved since this period with the omission of 5‐fluorouracil, increased use of paclitaxel and the use of more dose‐dense regimens, anthracyclines and taxanes as used here do still remain the mainstay of adjuvant breast cancer therapy, making these regimens still relevant to answer the question of the impact of early discontinuation. Dose‐dense chemotherapy was not clinically recommended practice in England during the study period; therefore, patients treated with dose‐dense schedules were not present in the dataset.^25^ Patients treated with SACT in either adjuvant/neoadjuvant settings were eligible. Patients treated with trastuzumab alongside chemotherapy were labelled, and this covariate was incorporated into survival analysis. Patients were excluded if they had no record of chemotherapy or other SACT, were treated with SACT as part of a clinical trial, were treated for a synchronous cancer or received hormone therapy alone without cytotoxic chemotherapy. Patients with missing date of death or date of death recorded before the end of SACT treatment in error were excluded. Stage 1 patients were not included in the data request due to only around 12% of Stage 1 patients receiving chemotherapy in the United Kingdom during the study period^28^ and were therefore not considered in this study. Hormone status was assigned using histological information available in the NCRD.

### 2.4 Treatment strategies

We compared the treatment strategies of completing greater than or equal to six cycles of chemotherapy to the reference group of treatment with less than six cycles in the included patients. Regimens were assigned based on records of individual drugs received. The regimens included in this study were typically given as six cycles during the study period, and therefore, we chose to include patients treated with these regimens only. Time of treatment discontinuation was defined as the last recorded first‐line chemotherapy administration.

### 2.5 Study outcomes

The study outcome was 5‐year all‐cause mortality. Patients were followed up for 5 years from chemotherapy initiation until the outcome of death, the last known follow‐up date or the end of the 5‐year follow‐up period. Patients were censored at the event of death or at the end of the 5‐year follow‐up period.

### 2.6 Covariates

Baseline covariates included age (at treatment initiation), timing of chemotherapy (whether chemotherapy was given in the adjuvant or neoadjuvant setting), body mass index (BMI), Charlson's comorbidity index,^29^ trastuzumab treatment, Index of Multiple Deprivation (IMD) quintiles for socio‐economic status,^30^ region of England, treating hospital type (Academic or General hospital), ethnicity group (Asian, Black, Mixed, Other, White or Unknown), tumour histology, cancer stage and chemotherapy regimen.

### 2.7 Statistical analysis and emulation of the target trial with the clone‐censor‐weight approach

Baseline characteristics were presented as mean (standard deviation [SD]) for continuous variables and as numbers (%) for categorical variables. Because of the cloning step of the clone‐censor‐weight approach, all baseline characteristics were the same between cohorts assigned to different treatment strategies. The baseline characteristics were reassessed at Months 6 and 12 post‐baseline before and after applying the inverse probability of censoring weights (IPCWs). Standardized mean difference (SMD) was used to evaluate the differences in baseline variables between groups. An SMD lower than 0.1 was considered a good balance between groups. Findings were considered to be statistically significant when the 95% confidence intervals (CIs) for risk on a relative scale did not cross 1 or when the 95% CI for risk difference on an absolute scale did not cross zero.

Target trial emulation with the clone‐censor‐weight approach has been recommended for investigating the efficacy and safety of cancer treatment strategies.^31^ We created a dataset with two copies of each eligible individual (i.e., cloning) and assigned each of the replicates to one of the treatment strategies at the start of follow‐up (time zero), that is, the date of receiving the first cycle of chemotherapy. We assessed whether patients adhered to their assigned treatment strategy at monthly intervals for 60 months; patients were censored if and when their actual treatment deviated from their assigned treatment strategy, thereby ensuring that patients followed their assigned strategy. That is, a patient clone who was assigned to the treatment strategy of less than six cycles of chemotherapy would be censored when they received the sixth cycle; a patient clone who was assigned to greater than or equal to six cycles would be censored when they discontinued treatment before the sixth cycle. Discontinuation was defined as the last record of chemotherapy or a period of >63 days from the last treatment cycle (accounting for the 3‐week interval between cycles plus a 6‐week grace period). Patients were censored at the end of this grace period for treatment deviation.

IPCW was then used to account for the selection bias from artificial censoring. The IPCW was calculated based on the probability of being uncensored that was estimated with logistic regression models. We fit two models, one for each treatment strategy arm, to allow treatment‐covariate interactions. The models predicted the monthly probability of being uncensored, including variables for time and the covariates as mentioned. All continuous variables (age, BMI and Charlson's comorbidity index) were modelled as restricted cubic splines with five knots. We then calculated the weights as 1/(probability of being uncensored). Theoretically, the weights created two pseudo‐populations in which treatment initiation was independent of measured prognostic factors. To avoid the influence of extreme weights, all weights are truncated at the 99th percentile.

Lastly, the replicated datasets with calculated weights are stacked and analysed with a weighted pooled logistic regression model. The model further included all baseline covariates, a treatment indicator, time from index (in linear and quadratic terms) and the interaction terms between time and treatment indicator. This model predicts the risk of the study outcome for each participant on each treatment strategy at each time interval. We can use these predicted risks to compute the population‐average cumulative risk (and risk difference and ratio) of the study outcome on treatment strategies at each monthly interval. This time‐discrete absolute risk of death was used to calculate the survival probability at each time and to plot the standardized, weighted survival curves. The 95% CIs for absolute risks and risk difference and ratios were calculated using a non‐parametric bootstrap of 300 samples from the main analysis. We also approximated hazard ratios (HRs) using odds ratios from a standardized, weighted pooled logistic regression and 95% CIs with the robust variance estimator, given that mortality is rare during each monthly follow‐up interval.^32^

**FIGURE 1:**
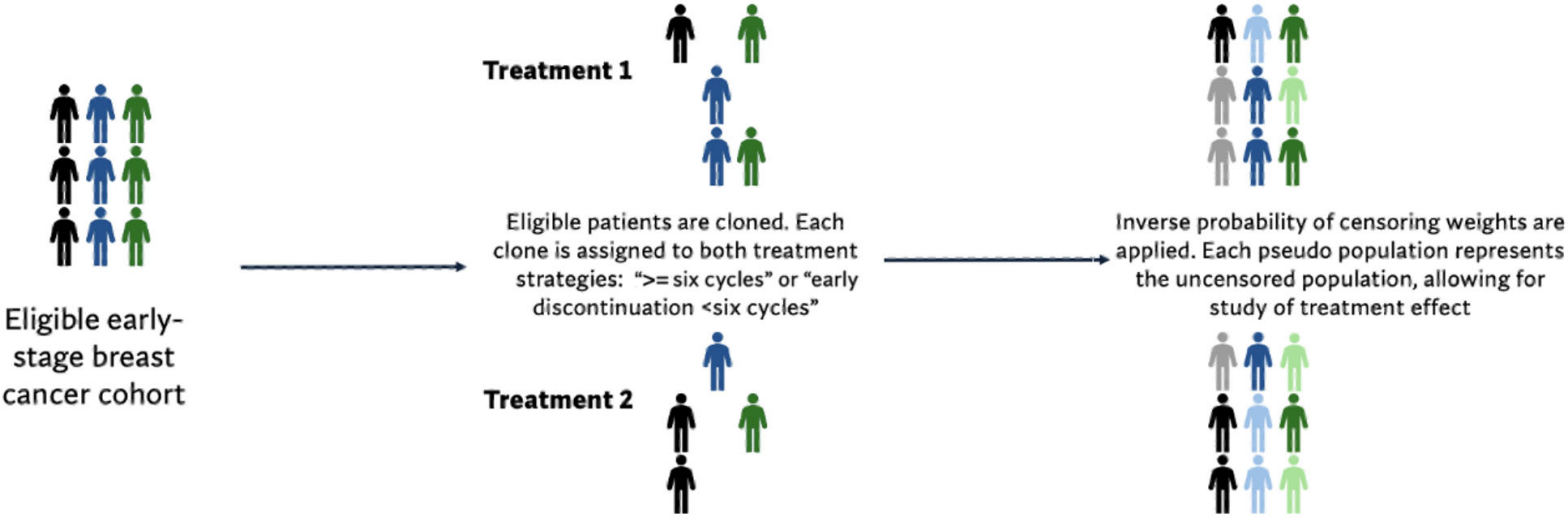
Schematic of the clone censor weight study design, showing cloning step and inverse probability of censoring weighting.

The study design is summarized in Figure 1.

### 2.8 Secondary analysis

For the secondary analysis, a similar clone‐censor‐weight approach was performed. Three copies of the patient cohort were created, and each was assigned to one treatment strategy. For example, a patient clone who was assigned to six cycles would be censored when they discontinued treatment before the sixth cycle, or when they received the seventh cycle; a patient clone who was assigned to greater than six cycles would be censored when they discontinued treatment before the seventh cycle. The weighting models were fit separately for these three treatment arms, and a standardized, weighted multinomial pooled logistic regression was used as the outcome model.

### 2.9 Subgroup and sensitivity analysis

We also conducted several pre‐specified subgroup and sensitivity analyses. Patient cohorts were stratified by cancer stage, histology, surgery type, age category (≥60 or <60 years), region, hospital type, obese/non‐obese status, ethnicity and index of multiple deprivation. Secondly, we repeated the analysis using untruncated IPCW. Thirdly, we conducted a complete case analysis by excluding patients with missing data in BMI, tumour histology or ethnicity.

We also present survival analysis using a standard, time‐fixed methodology to compare survival estimates with the target trial emulation and clone censor weight analytical approach. This approach assigned treatment strategy post‐baseline (i.e., using future information, which can introduce immortal time bias) and adjusted for confounding variables using regression adjustment models. Comparing these methodologies can allow an understanding of any biases associated with traditional, time‐fixed methodologies and how these are accounted for using the clone censor weight approach.

### 2.10 Missing data handling

The dataset was explored to understand the completeness of chemotherapy cycle records following the first cycle in the SACT dataset, a known limitation of this resource.^22^ Where patients had a period of >29 days between cycles, a missing cycle was imputed into the dataset at 21‐days from the previous cycle. Numbers of patients for whom missing cycles were imputed is described in the results. The intended (planned) number of cycles was not available in the datasets available, and detailed information on cancer diagnoses (i.e., number of nodes involved in disease) was also limited in the data sources. BMI was imputed for patients with no available height and weight data in the SACT dataset in order to provide a complete dataset for survival analysis. Multiple imputations by chained equations (MICE) was performed using 20 imputations as per to the standard methodology described by other authors.^33^ Missingness of data of other baseline covariates is described in Section 3. The incorporation of relative dose intensity into our analysis was explored; however, after thorough exploration of the data, we did not find a reliable methodology to identify patients who may have had their dose capped due to higher BSA, and therefore, we made the decision not to include dose intensity in our analysis.

Data handling was performed using RStudio v4.3, and analysis was performed using SAS version 9.4 (SAS Institute Inc).

### 2.11 Nomenclature of targets and ligands

Key protein targets and ligands in this article are hyperlinked to corresponding entries in.

http://www.guidetopharmacology.org, and are permanently archived in the Concise Guide to PHARMACOLOGY 2021/22.^34^

## 3 RESULTS

**FIGURE 2:**
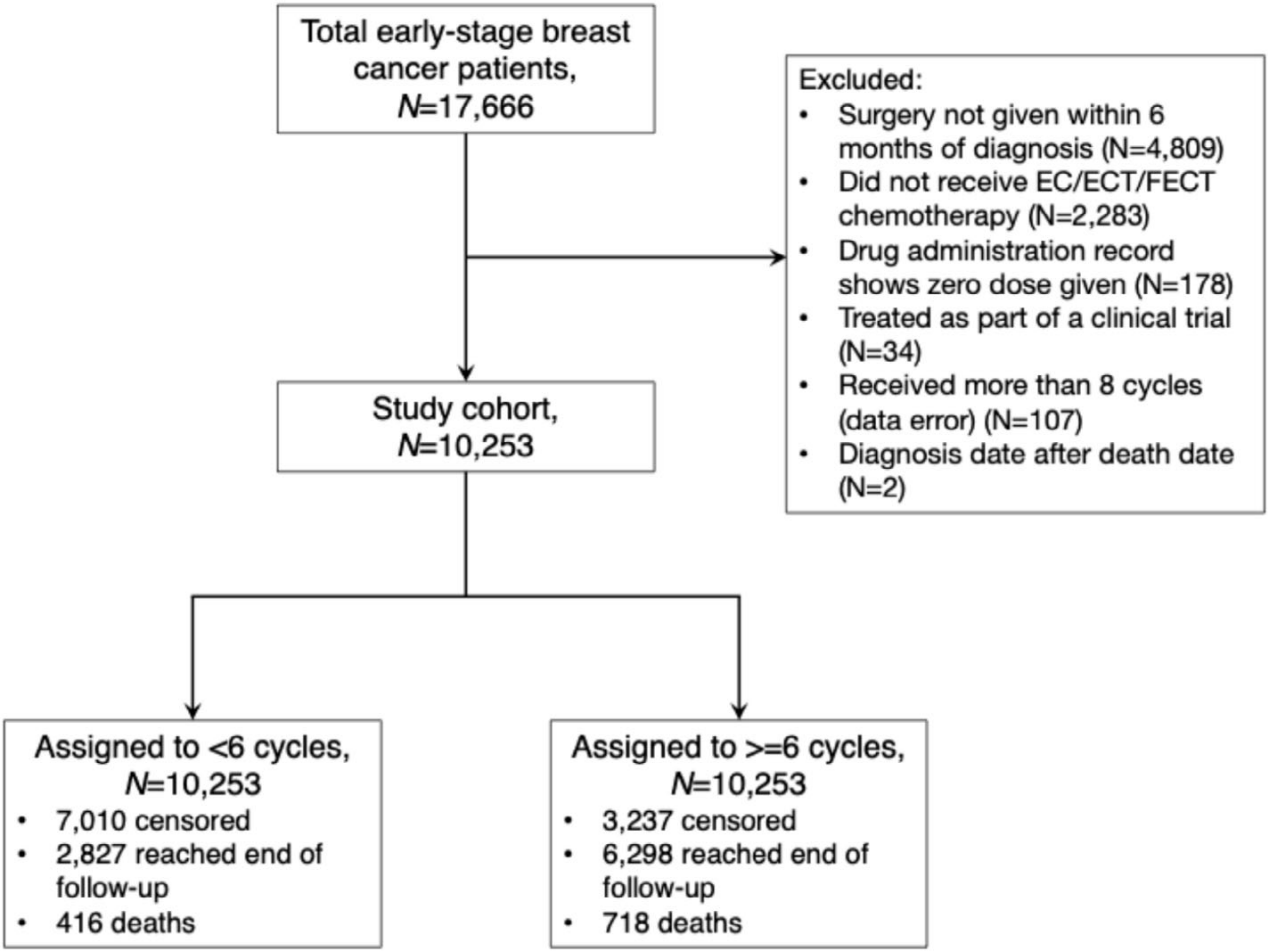
Selection of patients from the SACT dataset for trial emulation.

There were 17 666 patients treated for early breast cancer in the database in the 2‐year 2014–2015 period. 10 253 patients were included in the analysis cohort. 10 253 patients were excluded according to the criteria, shown as a flowchart (Figure 2).

Median age at start of chemotherapy treatment was 53.3 years. 39% received ECT (*n* = 4010), 35% EC (*n* = 3603) and 26% FECT (*n* = 2640). 75% of patients were diagnosed at Stage 2 (*n* = 7730) and 25% at Stage 3 (*n* = 2523). 82% of patients underwent surgery in combination with chemotherapy (74% adjuvant chemotherapy, *n* = 7554; 21% neoadjuvant, *n* = 2250). 4.4% did not have a record of surgery available (*n* = 449) but were included in the study cohort to avoid selection bias. The most common hormone status was ER+/HER2− (47%, *n* = 4855). All baseline characteristics are described in Table 2.

**TABLE 2:** Baseline characteristics of patients included in the trial emulation.

	All patients (*n* = 10 253)
Age, years (SD)	53.3 (10.9)
BMI, kg/m^2^ (SD)	28.3 (6.2)
Charlson's comorbidity index (SD)	0.1 (0.3)
Index of multiple deprivation (quintile, %)	
1—least deprived	2309 (22.5)
2	2311 (22.5)
3	1988 (19.4)
4	1862 (18.2)
5—most deprived	1783 (17.4)
Region (%)	
East of England	1272 (12.4)
London	1462 (14.3)
Midlands	1568 (15.3)
North East and Yorkshire	1692 (16.5)
North West	1776 (17.3)
South East	1644 (16)
South West	839 (8.2)
Centre type (%)	
Teaching hospital	7547 (73.6)
District general hospital	2706 (26.4)
Ethnicity (%)	
Asian	482 (4.7)
Black	316 (3.1)
Mixed	71 (0.7)
Other	170 (1.7)
White	8919 (87)
Unknown	295 (2.9)
Tumour histology (%)	
ER+/HER2+	1086 (10.6)
ER+/HER2−	4855 (47.4)
ER−/HER+	486 (4.7)
TNBC	1339 (13.1)
Unknown	2487 (24.3)
Tumour stage (%)	
2	49 (0.5)
2A	4425 (43.2)
2B	3256 (31.8)
3	22 (0.2)
3A	1726 (16.8)
3B	223 (2.2)
3C	552 (5.4)
Chemotherapy regimen (%)	
EC	3603 (35.1)
ECT	4010 (39.1)
FECT	2640 (25.7)
Previous trastuzumab treatment (%)	487 (4.7)
Surgery settings (%)	
Adjuvant	7554 (73.7)
Neoadjuvant	2250 (21.9)
No surgery	449 (4.4)

**FIGURE 3:**
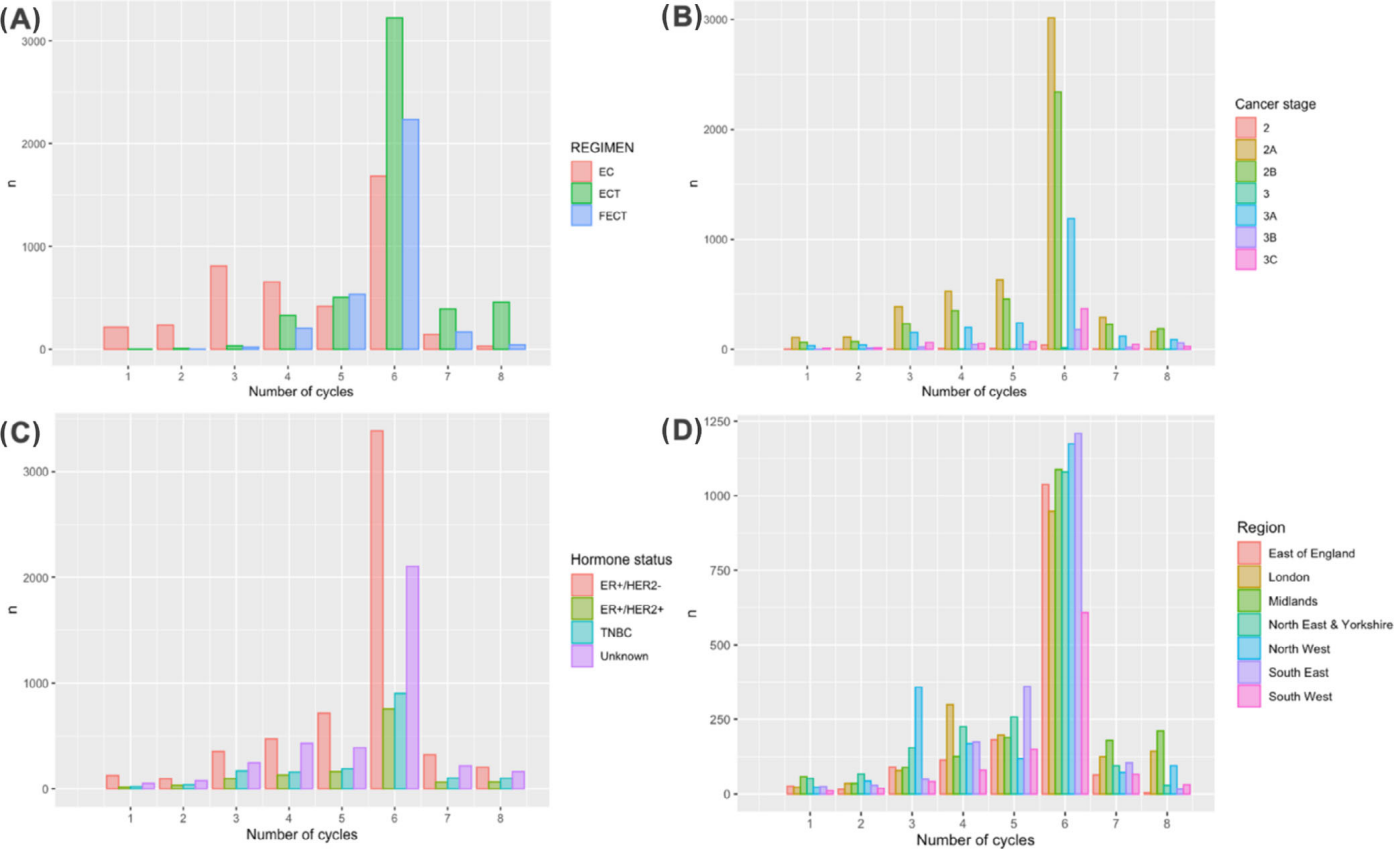
Number of cycles given by (A) regimen, (B) cancer stage at diagnosis, (C) hormone status and (D) English region.

The rate of early discontinuation increased with age, with 28% (95% CI 27, 29) in patients <60 years of age *vs*. 42% (95% CI 40, 44) in patients ≥60 years. Patients of normal BMI (18.5–25) discontinued chemotherapy at a lower rate (28%, 95% CI 26, 29) than overweight (32%, 95% CI 31, 34) and obese patients (35%, 95% CI 33, 36). The North East and Yorkshire showed the highest levels of early discontinuation (39%, 95% CI 36, 41) *vs*. 24% (95% CI 22, 27) in the Midlands. The rates of discontinuation did not differ significantly by cancer stage, hormone status or Index of Multiple Deprivation status (Figure 3).

### 3.1 Survival analysis of treatment effects

**FIGURE 4:**
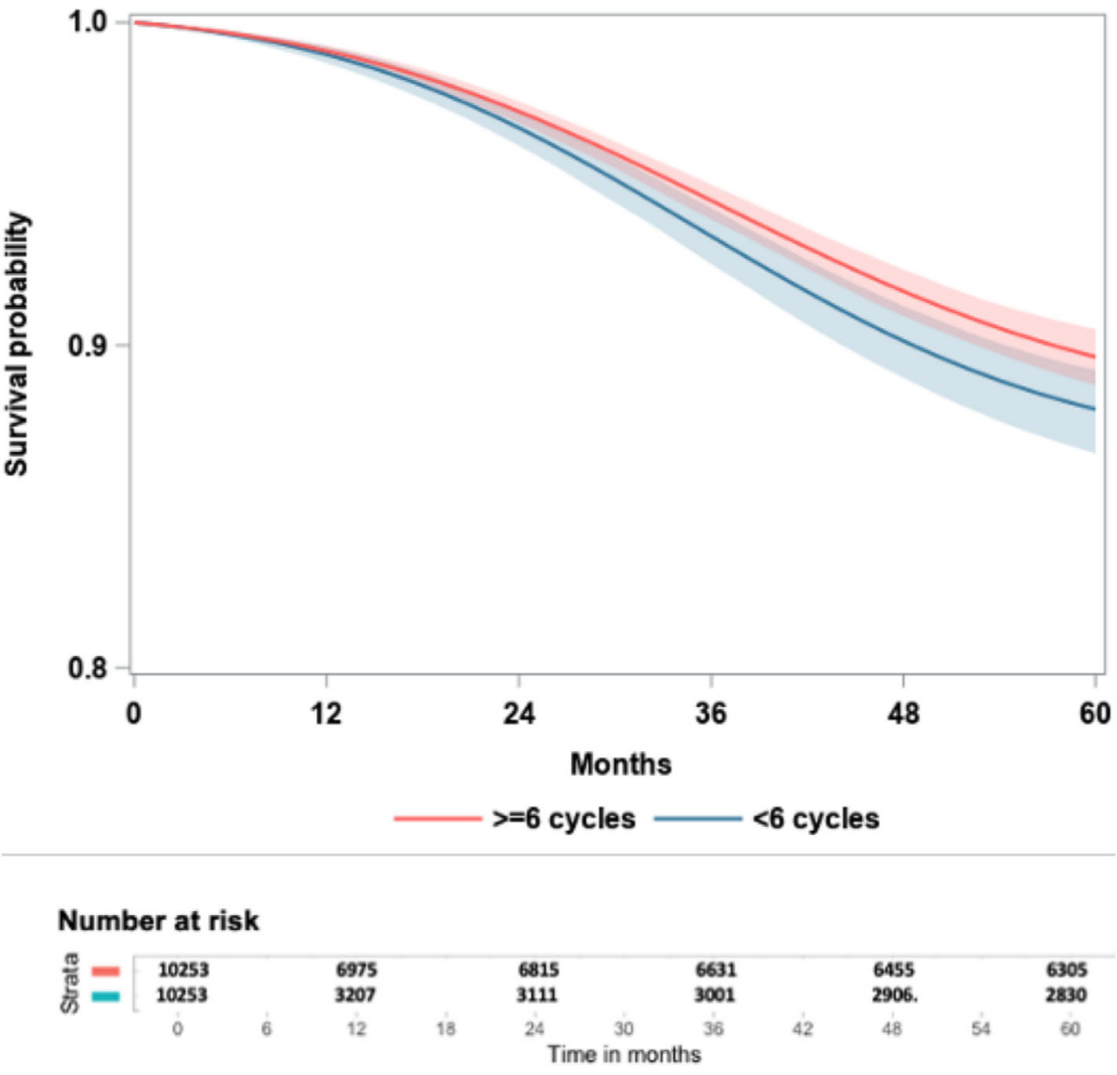
Survival probability comparing patients receiving greater than or equal to six cycles and less than six cycles of chemotherapy in patients with early‐stage breast cancer. 95% confidence intervals are shown by the shaded area. The number of patients at risk in each treatment group is shown in the table.

Each patient was followed up for 5 years from initiation of chemotherapy and was censored at the event of death or if they deviated from the assigned treatment strategy. The distributions of IPCW before and after truncation are presented in Table S1. After weighting, all baseline characteristics were well‐balanced between the two groups, measured at months 6 and 12 post‐baseline (Figure S1). Five‐year absolute risk, risk ratio and HRs were calculated to compare 5‐year all‐cause mortality between the treatment strategies of six cycles *vs*. less than six cycles. Five‐year mortality risk was 10.4% (95% CI 9.5, 11.2) in the greater than or equal to six cycles group compared with 12.0% (95% CI 10.8, 13.4) in the less than six cycles group. HR was 0.85 (95% CI 0.74, 0.98). The 5‐year survival curve is given in Figure 4, and the results of the survival analysis are in Table 3.

**TABLE 3:** Five‐year absolute risks, risk differences, risk ratios and hazard ratios for all‐cause mortality comparing receiving less than six cycles and greater than or equal to six cycles of chemotherapy in patients with early‐stage breast cancer.

Treatment	No. of patients	No. of outcomes	Follow‐up (person‐years)	5‐year absolute risk (%) (95% CI)	5‐year risk difference (%) (95% CI)	Hazard ratio (95% CI)
<6 cycles	10 253	416	18 190	12.0 (10.8, 13.4)	Reference	Reference
≥6 cycles	10 253	718	34 516	10.4 (9.5, 11.2)	−1.6 (−3.2, −0.1)	0.85 (0.74, 0.98)

### 3.2 Subgroup analyses

Subgroup analyses showed a significant survival benefit of treatment with greater than or equal to six cycles of chemotherapy in many subgroups. Patients with a stage 2 diagnosis showed a positive association of treatment with six cycles (HR 0.82, 95% CI 0.69, 0.98) relative to Stage 3 (HR 0.94, 95% CI 0.75, 1.18). Patients with TNBC and ER+/HER2− did not show improved survival when treated with greater than or equal to six cycles (TNBC HR 1.19, 95% CI 0.87, 1.62, ER+/HER2− HR 0.81, 95% CU 0.65, 1.00). Patients from the most deprived socio‐economic backgrounds (IMD quintile 4–5) showed superior survival when treated with six cycles (HR 0.79, 95% CI 0.64, 0.98) relative to those from less deprived backgrounds (HR 0.89, 95% CI 0.74, 1.07). Survival benefit was also observed when receiving six cycles for patients treated at general hospitals (HR 0.73, 95% CI 0.56, 0.94) and for non‐White patients (HR 0.56, 95% CI 0.34, 0.91). Results of all subgroup analyses are given in Table S2.

### 3.3 Sensitivity analysis

Complete‐case analysis and analysis with untruncated weight yield results are consistent with those from the main analysis (given in Tables S3 and S4).

### 3.4 Secondary analysis

A secondary analysis was performed to compare 5‐year survival between treatment strategies of exactly six cycles, greater than six cycles or less than six cycles. The flowchart for patient selection is presented in Figure S2. Hazard ratios were 0.84 (95% CI 0.72, 0.98) for six cycles and 0.84 (95% CI 0.61, 1.17) for those treated with greater than six cycles *vs*. less than six cycles (Table S5). Figure S3 shows the 5‐year survival curve stratified by these treatment strategies.

### 3.5 Comparison with time‐fixed methodology

Analysis using a standard, time‐fixed approach calculated a HR of 0.80 (95% CI 0.72, 0.92), adjusting for confounding variables. The survival curve is shown in Figure S4.

### 3.6 Missing data

A total of 1910 (19%) of patients were identified as having missing records of second cycles of chemotherapy, after checking SACT records against surgical records to ensure that gaps in treatment were not for surgery. For these patients, an extra cycle was imputed into the dataset. BMI data were available for 91.3% of patients before multiple imputation was performed.

## 4 DISCUSSION

This is the first study to investigate the impact of completing chemotherapy *vs*. early discontinuation on 5‐year all‐cause mortality in early‐stage breast cancer treated with curative intent, using a large population‐based cohort with near‐total coverage of the English patient population. Using a target trial emulation framework and clone‐censor‐weight analytical approach, findings presented here suggest a 5‐year survival benefit from completing greater than or equal to six cycles of chemotherapy compared with discontinuing early before receiving six cycles (HR 0.85, 95% CI 0.74, 0.98). Patients who discontinued chemotherapy before completing greater than or equal to six cycles had a 1.6% greater absolute risk of mortality within 5 years compared with those completing all six cycles. Early discontinuation occurred at higher rates in older patients, which may be explained by poorer tolerance of chemotherapy toxicity in patients ≥60 years. Additionally, the secondary analysis did not demonstrate improved survival for patients receiving greater than six cycles, although the estimate is highly imprecise (HR 0.84, 95% CI 0.61, 1.17) and is likely due to a limited sample size in this patient group.

Our findings are concordant with those of others investigating early chemotherapy discontinuation in other cancers. A comparable analysis in colorectal cancer found an HR of 0.49 (95% CI 0.38, 0.64) for 5‐year all‐cause mortality when completing all planned chemotherapy cycles.^15^ A HR of 1.51 (95% CI 1.31, 1.74) has also been reported for colorectal cancer patients treated with oxaliplatin receiving less than 75% of planned chemotherapy cycles.^35^ Our study found a significant negative effect of early chemotherapy discontinuation; however, the effect size observed in our study is significantly lower than both of these studies, which may be accounted for by employing the novel clone censor weight methodology to minimize for the bias of confounding, or the fact that chemotherapy is only one modality used to reduce mortality from breast cancer. The absolute risk difference of −1.6% in our analysis supports a small but significant association between early discontinuation of chemotherapy and increased risk of mortality, which has been reported in studies investigating this issue in pancreatic^10^ and colorectal cancers.^11^ In pancreatic cancer, early treatment discontinuation has been associated with poorer 2‐year survival with a larger effect size (HR 2.55, 95% CI 1.39, 4.68).^13^, ^36^ We acknowledge that these studies may be subject to time‐related bias that may result in larger risk estimates; however, associations of early treatment discontinuation with increased mortality have been consistently observed in multiple tumour types, concordant with our findings. Other studies have investigated the impact of early discontinuation of endocrine therapy in breast cancer.^18^, ^19^ Both of these studies used time‐varying approaches and identified an increased risk of mortality associated with early discontinuation of endocrine therapy (defined as discontinuation of endocrine before 4.5 years, or within 180 days of initiation, respectively). These results are consistent with our results for early discontinuation of chemotherapy; however, these studies investigated a different treatment modality in the adjuvant setting, a different exposure of interest and patient population.

Results of subgroup analyses suggest a differential benefit to receiving greater than or equal to six cycles between patients with different characteristics. The effect size of completing six cycles was significant in ER+/HER2+ patients, whereas other subgroups did not observe a significant benefit when receiving greater than or equal to six cycles. In particular, TNBC patients showed a HR of 1.19; however, the CIs crossed the null, and therefore, it cannot be concluded that treatment with greater than or equal to six cycles is associated with a significant survival benefit in the TNBC cohort. A significant finding from the subgroup analyses is that ‘non‐White’ ethnicity, relative to ‘White’, was associated with significantly improved survival when completing six cycles of chemotherapy (HR 0.56, 95% CI 0.34, 0.91), suggesting that ethnic minority patients can derive significantly improved survival benefit when completing greater than or equal to six cycles of chemotherapy.

Regional differences were observed in the number of patients completing six cycles of chemotherapy, with 39% of patients in the North East and Yorkshire receiving less than six cycles compared with 24% in the Midlands. Reasons for these disparities are complex and may partly relate to the clinician's preference in the intended number of planned cycles, according to flexibility in regimen choice and number of cycles given within European Society of Medical Oncology guidelines.^5^ Capacity of cancer treating centres to treat patients, variation in access to supportive care such as granulocyte‐colony stimulating factor (GCSF), and levels of patient education in the management of treatment‐related toxicity are all likely to affect the number of cycles of chemotherapy that patients are able to complete. Other work by our research group has identified variation between hospitals in the criteria used for giving chemotherapy, particularly in threshold values used for blood markers to decide whether the patient is fit for treatment.^37^

Given the small (but statistically significant) reduction in 5‐year survival observed in this study, clinicians should consider the rationale for chemotherapy discontinuation against the possible reduced treatment benefit. Continuous monitoring and effective management of treatment‐related toxicity, particularly in patients treated with anthracyclines, should be prioritized to allow as many patients as possible to complete all planned cycles.^38^ Other work in early‐stage breast cancer has found associations between greater burden of psychological comorbidities and treatment cessation before completing the planned number of cycles, suggesting that psychological well‐being may be an important factor in empowering patients to complete the planned number of chemotherapy cycles.^12^ A combination of pre‐emptive patient management, supportive medication and thorough patient education on toxicity management is therefore likely to allow a higher proportion of patients to complete the total number of planned cycles and achieve optimal survival outcomes in this cohort. Patient education in self‐management of toxicity is also an important factor, and patients should be educated in the importance of completing treatment as planned, given these findings.

### 4.1 Strengths and limitations

To our knowledge, this is the first study to use a target trial emulation approach to investigate the impact of discontinuing chemotherapy before receiving the standard planned number of cycles in early‐stage breast cancer. Observational studies of this nature are subject to time‐related biases, which we minimized using clone‐censor‐weight methodology to account for potential characteristic differences between patients completing greater than or equal to six or less than six cycles. We acknowledge the possibility for unmeasured confounding; however, our study design was carefully considered to adequately control for confounding and time‐related biases, and the major factors affecting survival that are measurable, such as cancer stage, hormone status, age and other demographic factors, have been captured and incorporated into our study, therefore providing sufficient information for a reliably specified survival model.

In comparison with the time‐fixed analytical approach, the HR calculated was similar (HR 0.80, 95% CI 0.72, 0.92). The small difference observed here (with a larger effect size, illustrated by the early separation of survival curves in favour of the greater than or equal to six cycles treatment group), shows a small degree of immortal time bias using the time‐fixed approach. The extent of this is small, due to the relatively low mortality rate, whereas patients are on active treatment for early‐stage breast cancer. Greater bias would be expected in cancer types where mortality is greater and death during active treatment occurs more commonly, such as advanced lung cancer; however, in this study, the effect of immortal time bias was small.

Within our cohort, there was variation in clinical practice both within and between regions, which likely reflects differences in clinical practice, for both regimens and duration of treatment. This feature of clinical practice presented some difficulty in defining ‘completion’ of chemotherapy when designing our study. Other research has used thresholds of ‘<80% of planned cycles’^12^ or ‘failing to complete all planned cycles’^39^ as definitions of early treatment discontinuation; however, the datasets used for our analysis do not provide information on the planned number of cycles, so we could not use this definition. We accounted for the difficulty in defining ‘full’ treatment by prioritizing exploration of the dataset in the preliminary design phase, investigating the number of chemotherapy received by patient demographics and region, which has been presented and discussed. Clinicians have highlighted that factors such as age, hormone status and comorbidity burden will guide the intended number of cycles given. The number of cycles received may also be related to prognosis; for example, patients treated with six cycles may represent a group with greater disease burden, where more cycles are given to maximize control of disease, whereas those completing less cycles may be less fit, older and suffer greater comorbidity or may have less extensive disease which the clinician believes can be controlled with fewer chemotherapy cycles. The intended number of chemotherapy cycles was not available in the datasets; however, we accounted for this and other potential differences in patient characteristics by using SMDs and the clone‐censor‐weight analytical approach to appropriately adjust for any differences in treatment intent in our analysis. This estimate provides a measurement of the per‐protocol effect of receiving six full cycles of treatment compared with less than six, based on the actual number of cycles the patients have received. Although such estimates do not represent the effect of treatment strategies with imperfect adherence under real‐world settings, they address a clinically relevant question regarding the consequences of completing a planned chemotherapy regimen. In oncology practice, treatment discontinuation and dose modification are common, and understanding the outcomes associated with completing treatment among patients who are able to adhere is informative for clinical decision‐making and patient counselling. As such, although the estimated effect should not be interpreted as a policy effect, it provides valuable insight into the potential benefits of completing a given number of chemotherapy cycles.

We would like to reiterate that the data available for this study were Cancer Registry and SACT dataset records, which do not contain laboratory, imaging, information on treatment response or specified comorbidities; therefore, these time‐varying covariates could not be incorporated into the analysis, and we have discussed this limitation in previous similar publications.^23^ Although incorporating these time‐varying covariates would be beneficial to this project, it was not possible in this analysis, given the data available.

## 5 CONCLUSIONS

This observational study showed that early discontinuation of chemotherapy was associated with a 1.6% increased risk of 5‐year mortality. The finding should be considered by clinicians when planning treatment to maximize treatment benefit from chemotherapy. The use of target trial emulation and clone‐censor‐weight methodologies can support future research into chemotherapy de‐escalation, in the absence of expensive and time‐consuming clinical trials.

## AUTHOR CONTRIBUTIONS

**Luke Steventon:** Project management; study design; data cleaning analysis; interpretation; manuscript drafting. **Chengsheng Ju:** Study design; data analysis; manuscript drafting and editing. **Kenneth K. C. Man:** Project oversight; study design; manuscript editing. **Rebecca Roylance:** Clinical oversight; manuscript editing. **Diego Ottaviani:** Clinical oversight; manuscript editing. **Chiara Creed:** Clinical oversight; manuscript editing. **Michal Sladkowski:** Clinical oversight; manuscript editing. **Alec Wisner:** Clinical oversight; manuscript editing. **Li Wei:** Supervision of project; manuscript editing. **Pinkie Chambers:** Project inception; supervision of project; clinical oversight; study design; manuscript editing.

## CONFLICT OF INTEREST STATEMENT

Chiara Creed declares research funding from Gilead, Novartis and B. Braun. Pinkie Chambers declares research funding from Gilead and Pfizer and an education grant from AbbVie. Kenneth K. C. Man declares grants from the Hong Kong Research Grant Council, the CW Maplethorpe Fellowship, UK National Institute for Health and Care Research and European Commission Framework Horizon 2020, Innovation and Technology Commission of the Government of the Hong Kong Special Administrative Region and personal fees from IQVIA Ltd. Michal Sladkowski declares Honoraria from Novartis and Roche. Luke Steventon, Chengsheng Ju, Rebecca Roylance, Diego Ottaviani, Alec Wisner and Li Wei declare no conflict of interest.

## PATIENT AND PUBLIC INVOLVEMENT STATEMENT

Patients were involved in the design, interpretation and reporting of our findings. We initially discussed chemotherapy side effects with a patient focus group, who highlighted that the effects of discontinuing chemotherapy on cancer outcomes were unknown and concerned some patients. We then decided to investigate this issue through the PRUK grant, including patients in the application for funding. Throughout the project, we met with patients to discuss our findings and hear their perspective on the small reduction in survival outcomes that we observed was associated with early chemotherapy discontinuation. This guided the writing of the manuscript, particularly in the discussion section, where we highlighted various causes and reasons for early chemotherapy discontinuation and how this could be communicated effectively to patients.

## Supporting information


**Table S1.** Inverse probability weight distribution before and after truncation.
**Figure S1.** Balance of covariates before and after weighting at Month 6 and 12 post‐baseline.
**Table S2.** Subgroup analysis: Five‐year absolute risks, risk differences, risk ratios and hazard ratios for all‐cause mortality comparing receiving less than six cycles and greater than or equal to six cycles of chemotherapy in patients with early‐stage breast cancer.
**Table S3.** Untruncated weight: Five‐year absolute risks, risk differences, risk ratios and hazard ratios for all‐cause mortality comparing receiving less than six cycles and greater than or equal to six cycles of chemotherapy in patients with early‐stage breast cancer.
**Table S4.** Complete case analysis: Five‐year absolute risks, risk differences, risk ratios and hazard ratios for all‐cause mortality comparing receiving less than six cycles and greater than or equal to six cycles of chemotherapy in patients with early‐stage breast cancer.
**Figure S2.** Secondary analysis: Selection of patients from the SACT dataset for the 3‐arm trial emulation.
**Figure S3.** Secondary analysis: Survival probability comparing patients receiving less than six cycles, six cycles and greater than six cycles of chemotherapy in patients with early‐stage breast cancer. 95% confidence intervals are shown by the shaded area.
**Table S5.** Secondary analysis: Five‐year absolute risks, risk differences, risk ratios and hazard ratios for all‐cause mortality comparing receiving less than six cycles, six cycles and greater than six cycles of chemotherapy in patients with early‐stage breast cancer.

## Data Availability

The data that support the findings of this study are available from NHS Digital. Restrictions apply to the availability of these data, which were used under license for this study. Data are available from the authors with the permission of NHS Digital.
