# Supplementary material for: Completion vs. early discontinuation of chemotherapy and the impact on 5‐year all‐cause mortality in women treated for early‐stage breast cancer from 2015 to 2020: A cohort study using a target trial emulation approach
Source: Br J Clin Pharmacol. 2026 Mar 8;92(7):2272–83. doi: 10.1002/bcp.70488 (PMC13304275; doi:10.1002/bcp.70488)
Supplement: Supplementary file 1 — Table S1. Inverse probability weight distribution before and after truncation. Figure S1. Balance of covariates before and after weighting at Month 6 and 12 post‐baseline. Table S2. Subgroup analysis: Five‐year absolute risks, risk differences, risk ratios and hazard ratios for all‐cause mortality comparing receiving less than six cycles and greater than or equal to six cycles of chemotherapy in patients with early‐stage breast cancer. Table S3. Untruncated weight: Five‐year absolute risks, risk differences, risk ratios and hazard ratios for all‐cause mortality comparing receiving less than six cycles and greater than or equal to six cycles of chemotherapy in patients with early‐stage breast cancer. Table S4. Complete case analysis: Five‐year absolute risks, risk differences, risk ratios and hazard ratios for all‐cause mortality comparing receiving less than six cycles and greater than or equal to six cycles of chemotherapy in patients with early‐stage breast cancer. Figure S2. Secondary analysis: Selection of patients from the SACT dataset for the 3‐arm trial emulation. Figure S3. Secondary analysis: Survival probability comparing patients receiving less than six cycles, six cycles and greater than six cycles of chemotherapy in patients with early‐stage breast cancer. 95% confidence intervals are shown by the shaded area. Table S5. Secondary analysis: Five‐year absolute risks, risk differences, risk ratios and hazard ratios for all‐cause mortality comparing receiving less than six cycles, six cycles and greater than six cycles of chemotherapy in patients with early‐stage breast cancer. [file BCP-92-2272-s001.doc]

**Supplement**

**Table S1**. Inverse probability weight distribution before and after truncation.

|  | **Before truncation** | | **After truncation** | |
| --- | --- | --- | --- | --- |
| **Measurement** | <6 cycles | >=6 cycles | <6 cycles | >=6 cycles |
| 1^st^ percentile | 1.00 | 1.00 | 1.00 | 1.00 |
| 50^th^ percentile | 1.92 | 1.24 | 1.99 | 1.24 |
| 75^th^ percentile | 4.17 | 1.43 | 4.19 | 1.43 |
| 90^th^ percentile | 5.87 | 2.12 | 6.04 | 2.12 |
| 95^th^ percentile | 6.83 | 2.51 | 6.94 | 2.51 |
| 99^th^ percentile | 9.17 | 3.56 | 9.52 | 3.56 |
| Max | 149.87 | 13.17 | 9.52 | 3.56 |
| Mean | 2.85 | 1.44 | 2.89 | 1.44 |
| SD | 2.06 | 0.54 | 2.01 | 0.49 |

SD, standard deviation.


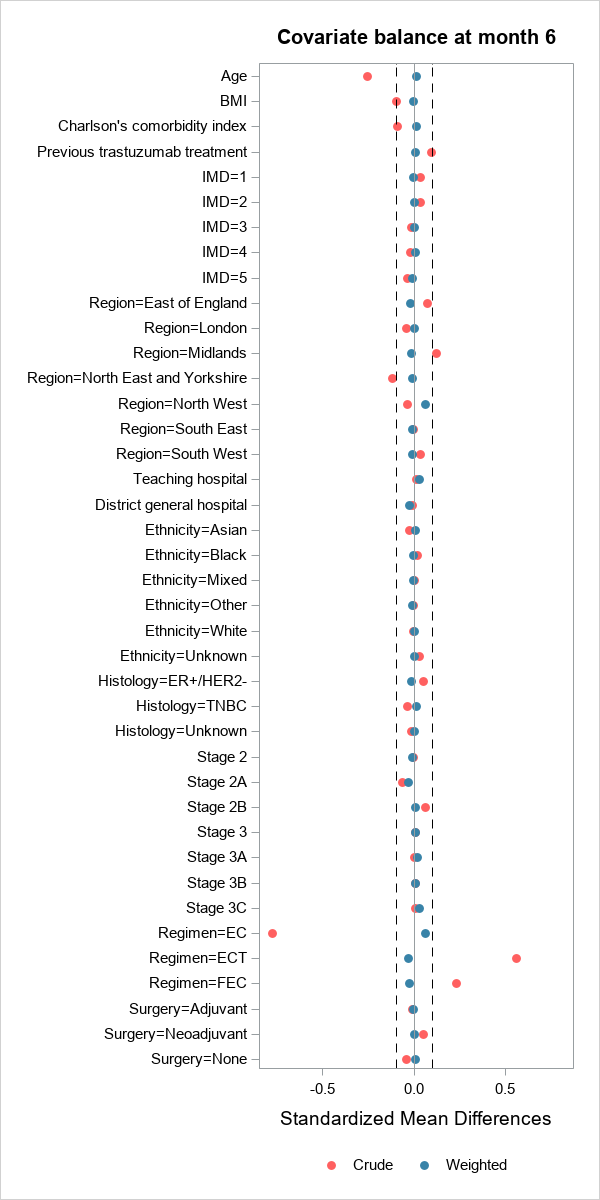

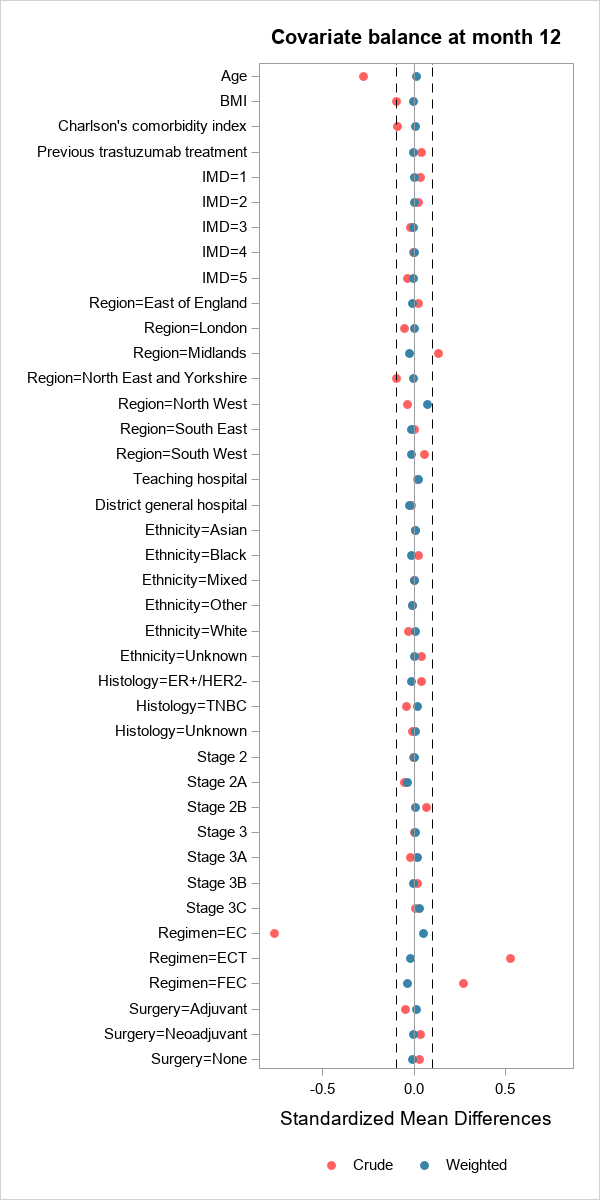


**Figure S1. Balance of covariates before and after weighting at month 6 and 12 postbaseline.**

**Table S2. Subgroup analysis:** **Five-year absolute risks, risk differences, risk ratios, and hazard ratios for all-cause mortality comparing receiving <six cycles, and >=six cycles of chemotherapy** of chemotherapy in patients with early-stage breast cancer.

| **Treatment** | **No. of patients** | **No. of outcomes** | **5-year absolute risk (%)** | **5-year risk difference (%)** | **Hazard ratio**  **(95% CI)** |
| --- | --- | --- | --- | --- | --- |
| **Age < 60 year** |  |  |  |  |  |
| <6 cycles | 7,151 | 210 | 10.5 | Reference | Reference |
| >=6 cycles | 7,151 | 476 | 8.9 | -1.5 | 0.84 (0.69 to 1.00) |
| **Age >= 60 years** |  |  |  |  |  |
| <6 cycles | 3,102 | 206 | 15.5 | Reference | Reference |
| >=6 cycles | 3,102 | 242 | 13.3 | -2.2 | 0.84 (0.68 to 1.03) |
| **Obese** |  |  |  |  |  |
| <6 cycles | 3,303 | 155 | 12.9 | Reference | Reference |
| >=6 cycles | 3,303 | 243 | 11.3 | -1.6 | 0.85 (0.68 to 1.07) |
| **Non-obese** |  |  |  |  |  |
| <6 cycles | 6,950 | 261 | 11.4 | Reference | Reference |
| >=6 cycles | 6,950 | 475 | 9.9 | -1.5 | 0.86 (0.72 to 1.03) |
| **IMD 1-3** |  |  |  |  |  |
| <6 cycles | 6,608 | 232 | 10.8 | Reference | Reference |
| >=6 cycles | 6,608 | 433 | 9.7 | -1.1 | 0.89 (0.74 to 1.07) |
| **IMD 4-5** |  |  |  |  |  |
| <6 cycles | 3,645 | 184 | 14.1 | Reference | Reference |
| >=6 cycles | 3,645 | 285 | 11.5 | -2.6 | 0.79 (0.64 to 0.98) |
| **Teaching hospital** |  |  |  |  |  |
| <6 cycles | 7,547 | 292 | 11.2 | Reference | Reference |
| >=6 cycles | 7,547 | 512 | 10.1 | -1.1 | 0.89 (0.76 to 1.06) |
| **District general hospital** |  |  |  |  |  |
| <6 cycles | 2,706 | 124 | 14.5 | Reference | Reference |
| >=6 cycles | 2,706 | 206 | 11.0 | -3.5 | 0.73 (0.56 to 0.94) |
| **White ethnicity** |  |  |  |  |  |
| <6 cycles | 8,919 | 371 | 12.0 | Reference | Reference |
| >=6 cycles | 8,919 | 646 | 10.7 | -1.3 | 0.88 (0.76 to 1.02) |
| **Non-White ethnicity** |  |  |  |  |  |
| <6 cycles | 1,334 | 45 | 13.2 | Reference | Reference |
| >=6 cycles | 1,334 | 72 | 7.8 | -5.4 | 0.56 (0.34 to 0.91) |
| **ER+/HER2+** |  |  |  |  |  |
| <6 cycles | 1,086 | 32 | 6.4 | Reference | Reference |
| >=6 cycles | 1,086 | 22 | 3.1 | -3.4 | 0.46 (0.24 to 0.90) |
| **ER+/HER2-** |  |  |  |  |  |
| <6 cycles | 4,855 | 165 | 10.7 | Reference | Reference |
| >=6 cycles | 4,855 | 290 | 8.8 | -1.9 | 0.81 (0.65 to 1.00) |
| **ER-/HER+** |  |  |  |  |  |
| <6 cycles | 486 | 20 | 11.3 | Reference | Reference |
| >=6 cycles | 486 | 31 | 8.7 | -2.5 | 0.75 (0.37 to 1.53) |
| **TNBC** |  |  |  |  |  |
| <6 cycles | 1,339 | 91 | 19.4 | Reference | Reference |
| >=6 cycles | 1,339 | 203 | 22.8 | 3.4 | 1.19 (0.87 to 1.62) |
| **Unknown** |  |  |  |  |  |
| <6 cycles | 2,487 | 108 | 13.4 | Reference | Reference |
| >=6 cycles | 2,487 | 172 | 10.0 | -3.4 | 0.73 (0.55 to 0.96) |
| **Stage 2** |  |  |  |  |  |
| <6 cycles | 7,681 | 247 | 9.4 | Reference | Reference |
| >=6 cycles | 7,681 | 409 | 7.8 | -1.6 | 0.82 (0.69 to 0.98) |
| **Stage 3** |  |  |  |  |  |
| <6 cycles | 2,572 | 169 | 19.1 | Reference | Reference |
| >=6 cycles | 2,572 | 309 | 18.5 | -0.7 | 0.94 (0.75 to 1.18) |
| **Regimen EC** |  |  |  |  |  |
| <6 cycles | 3,603 | 250 | 11.7 | Reference | Reference |
| >=6 cycles | 3,603 | 134 | 8.7 | -3.0 | 0.72 (0.57 to 0.90) |
| **Regimen ECT** |  |  |  |  |  |
| <6 cycles | 4,010 | 86 | 11.2 | Reference | Reference |
| >=6 cycles | 4,010 | 362 | 11.0 | -0.2 | 1.00 (0.78 to 1.29) |
| **Regimen FEC** |  |  |  |  |  |
| <6 cycles | 2,640 | 80 | 13.3 | Reference | Reference |
| >=6 cycles | 2,640 | 222 | 10.9 | -2.4 | 0.79 (0.60 to 1.04) |
| **Adjuvant** |  |  |  |  |  |
| <6 cycles | 7,554 | 285 | 10.5 | Reference | Reference |
| >=6 cycles | 7,554 | 466 | 9.3 | -1.2 | 0.87 (0.74 to 1.03) |
| **Neoadjuvant** |  |  |  |  |  |
| <6 cycles | 2,250 | 119 | 18.0 | Reference | Reference |
| >=6 cycles | 2,250 | 230 | 14.6 | -3.5 | 0.77 (0.59 to 1.01) |
| **No surgery** |  |  |  |  |  |
| <6 cycles | 449 | 12 | 11.1 | Reference | Reference |
| >=6 cycles | 449 | 22 | 7.1 | -3.9 | 0.59 (0.23 to 1.53) |

CI, confidence interval.

**Table S3. Untruncated weight: Five-year absolute risks, risk differences, risk ratios, and hazard ratios for all-cause mortality comparing receiving <six cycles, and >=six cycles of chemotherapy of chemotherapy in patients with early-stage breast cancer.**

| **Treatment** | **No. of patients** | **No. of outcomes** | **5-year absolute risk (%)** | **5-year risk difference (%)** | **Hazard ratio**  **(95% CI)** |
| --- | --- | --- | --- | --- | --- |
| <6 cycles | 10,253 | 416 | 12.0 | Reference | Reference |
| >=6 cycles | 10,253 | 718 | 10.4 | -1.6 | 0.85 (0.74 to 0.98) |

CI, confidence interval.

**Table S4. Complete case analysis: Five-year absolute risks, risk differences, risk ratios, and hazard ratios for all-cause mortality comparing receiving <six cycles, and >=six cycles of chemotherapy** of chemotherapy in patients with early-stage breast cancer.

| **Treatment** | **No. of patients** | **No. of outcomes** | **5-year absolute risk (%)** | **5-year risk difference (%)** | **Hazard ratio**  **(95% CI)** |
| --- | --- | --- | --- | --- | --- |
| <6 cycles | 6,889 | 279 | 11.9 | Reference | Reference |
| >=6 cycles | 6,889 | 496 | 10.7 | -1.2 | 0.90 (0.75 to 1.06) |

CI, confidence interval.


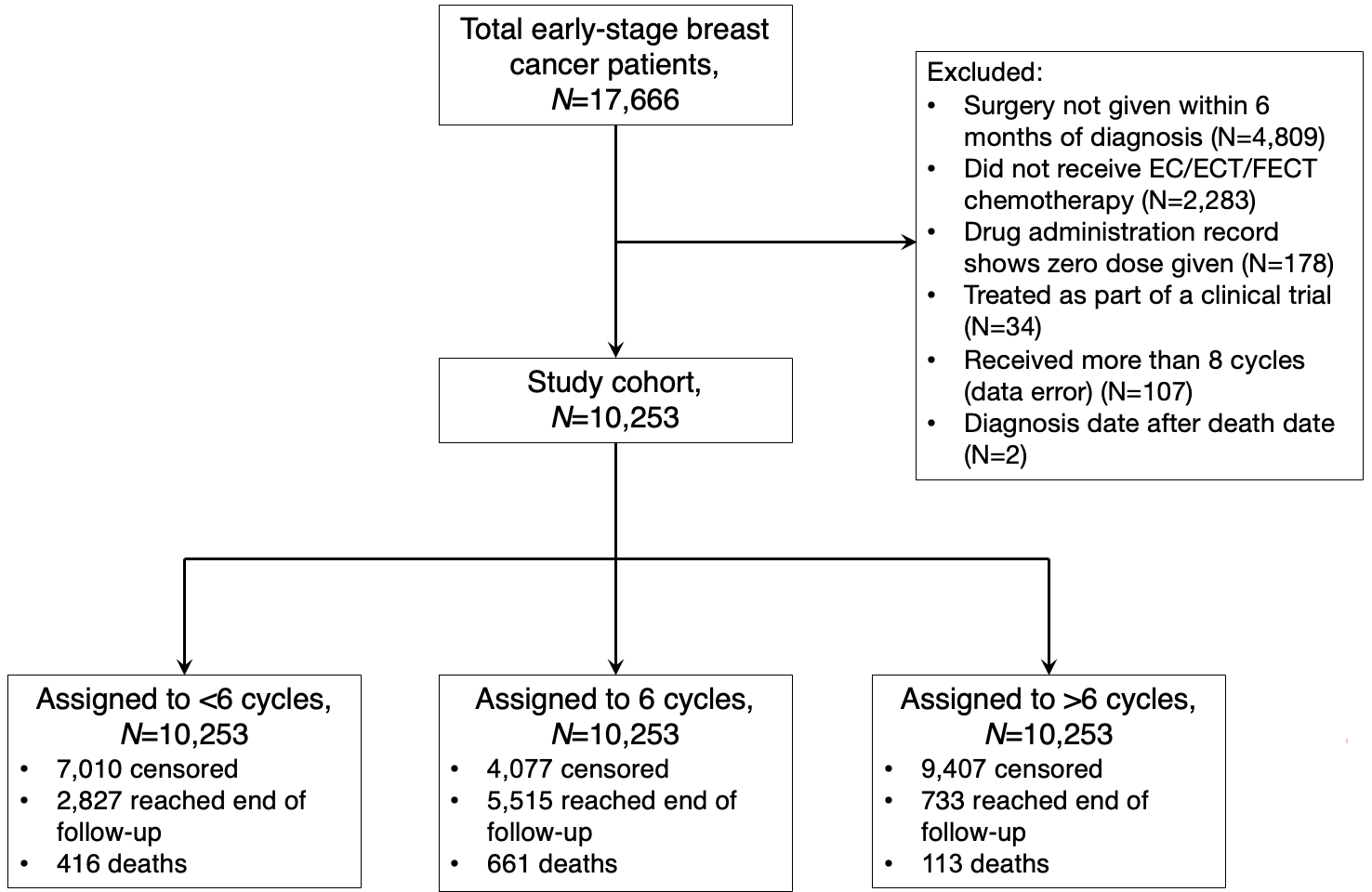


**Figure S2. Secondary analysis: Selection of patients from SACT dataset for the 3-arm trial emulation.**


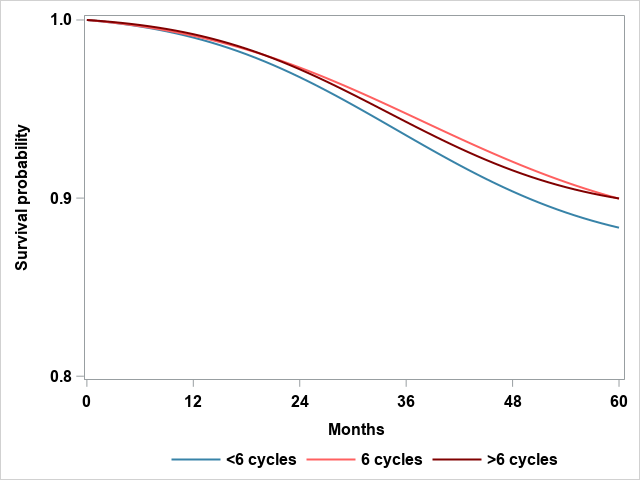


**Figure S3. Secondary analysis: Survival probability comparing patients** **receiving <six cycles, six cycles, and >six cycles of chemotherapy in patients with early-stage breast cancer. 95% confidence intervals are shown by the shaded area.**

**Table S5. Secondary analysis: Five-year absolute risks, risk differences, risk ratios, and hazard ratios for all-cause mortality comparing receiving <six cycles, six cycles, and >six cycles of chemotherapy** of chemotherapy in patients with early-stage breast cancer.

| **Treatment** | **No. of patients** | **No. of outcomes** | **5-year absolute risk (%)** | **5-year risk difference (%)** | **Hazard ratio**  **(95% CI)** |
| --- | --- | --- | --- | --- | --- |
| <6 cycles | 10,253 | 416 | 11.7 | Reference | Reference |
| 6 cycles | 10,253 | 611 | 10.0 | -1.6 | 0.84 (0.72 to 0.98) |
| >6 cycles | 10,253 | 113 | 10.0 | -1.6 | 0.84 (0.61 to 1.17) |
